# Host-mediated RNA editing in viruses

**DOI:** 10.1186/s13062-023-00366-w

**Published:** 2023-03-28

**Authors:** Tongtong Zhu, Guangyi Niu, Yuansheng Zhang, Ming Chen, Chuan-Yun Li, Lili Hao, Zhang Zhang

**Affiliations:** 1 National Genomics Data Center & CAS Key Laboratory of Genome Sciences and Information, Beijing Institute of Genomics, Chinese Academy of Sciences, Beijing, 100101 China; 2 China National Center for Bioinformation, Beijing, 100101 China; 3 University of Chinese Academy of Sciences, Beijing, 100049 China; 4 Laboratory of Bioinformatics and Genomic Medicine, Beijing Key Laboratory of Cardiometabolic Molecular Medicine, Institute of Molecular Medicine, Peking University, Beijing, 100871 China

**Keywords:** Virus, RNA editing, Deamination, Proviral, Antiviral

## Abstract

Viruses rely on hosts for life and reproduction, cause a variety of symptoms from common cold to AIDS to COVID-19 and provoke public health threats claiming millions of lives around the globe. RNA editing, as a crucial co-/post-transcriptional modification inducing nucleotide alterations on both endogenous and exogenous RNA sequences, exerts significant influences on virus replication, protein synthesis, infectivity and toxicity. Hitherto, a number of host-mediated RNA editing sites have been identified in diverse viruses, yet lacking a full picture of RNA editing-associated mechanisms and effects in different classes of viruses. Here we synthesize the current knowledge of host-mediated RNA editing in a variety of viruses by considering two enzyme families, viz., ADARs and APOBECs, thereby presenting a landscape of diverse editing mechanisms and effects between viruses and hosts. In the ongoing pandemic, our study promises to provide potentially valuable insights for better understanding host-mediated RNA editing on ever-reported and newly-emerging viruses.

## Introduction

RNA editing, as a crucial co-/post-transcriptional modification inducing nucleotide alterations on both endogenous and exogenous RNA sequences [1–3], plays important roles in many biological processes in nearly all forms of cellular life [4–7] and is associated with a variety of human diseases, including cancers [8, 9]. In metazoans, primary RNA editing types are adenosine to inosine (A-to-I; inosine is further recognized as guanosine) and cytidine to uridine (C-to-U), which are catalyzed by adenosine deaminases acting on RNA family (ADARs, including ADAR1, ADAR2, and ADAR3) and apolipoprotein B mRNA editing catalytic polypeptide-like family (APOBECs, including APOBEC1, APOBEC2, APOBEC3A-H, APOBEC4, and AID), respectively [4, 8, 10–15]. In addition to deamination activity, ADARs and APOBECs, also acting as RNA binding proteins, could interact with RNA targets directly and thus exert extra complicated molecular effects on biological functions of both endogenous and exogenous RNAs, especially virus RNAs [16–18].

**Fig. 1:**
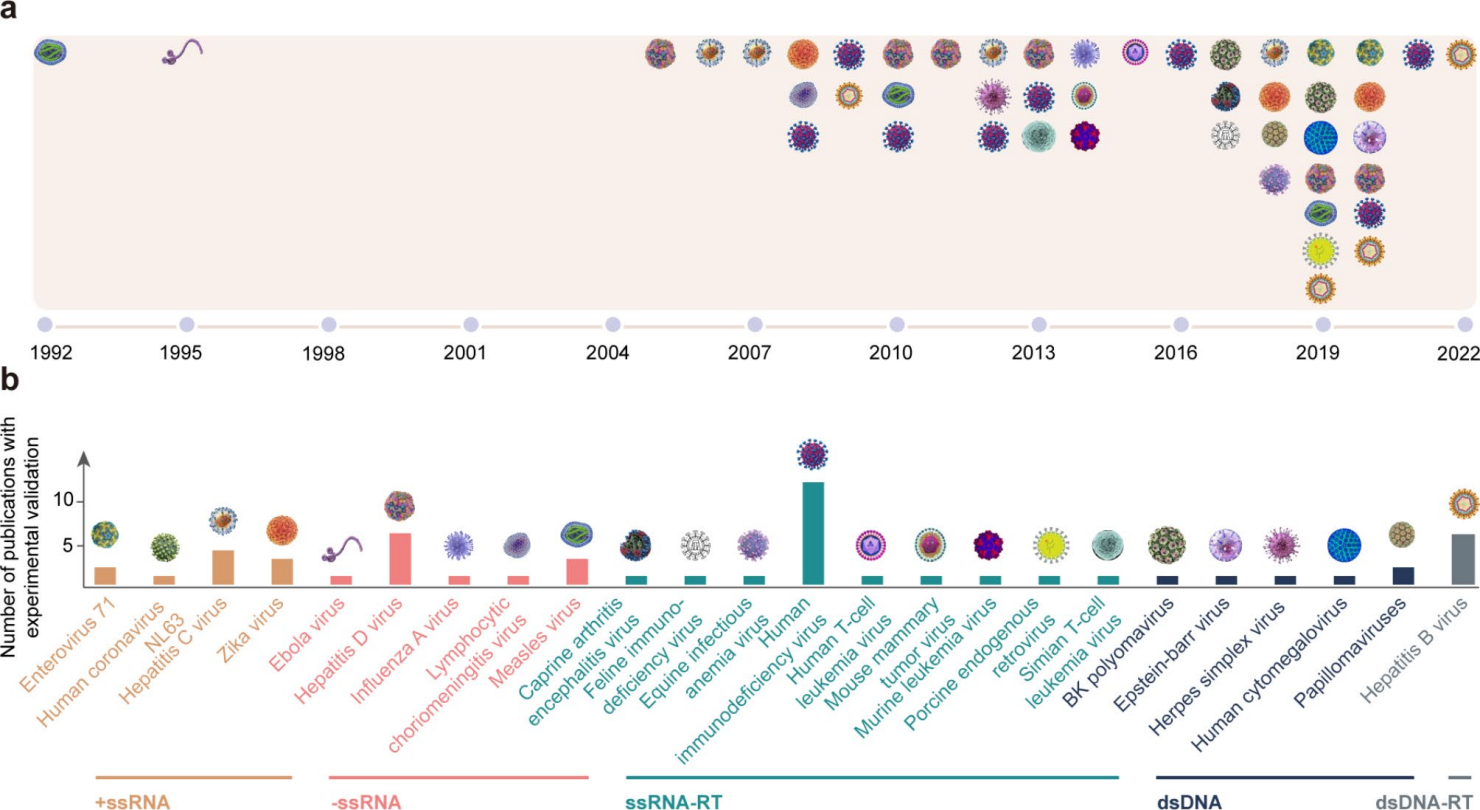
RNA editing events in viruses. **(a)** The timeline of host-mediated RNA editing events identified in viruses with experimental validation. **(b)** Number of experimentally validated publications in different classes of viruses

Viruses rely on hosts for life and reproduction, cause a variety of symptoms from common cold to AIDS to COVID-19 and provoke public health threats claiming millions of lives around the globe. Over the past decades, a number of studies have experimentally revealed complicated mechanisms between various viruses and their hosts mediated by RNA editing (Fig. 1). A case in point is the first discovery reported in 1992 that host-mediated RNA editing regulates the packaging and inhibits the replication of hepatitis D virus [19]. Based on the deaminase roles during the process of virus infection, the interaction between RNA editing enzymes and viruses can be classified into two categories, namely, deamination-dependent (cis-regulation) and deamination-independent (trans-regulation) [20]. For the former, ADARs and APOBECs catalyze the hydrolytic deamination directly on viral RNA substrates [21], which can provoke viral RNA sequence variations [22], changes of viral RNA secondary structures [73], and amino acid recoding [23]. For the latter, ADARs and APOBECs can be involved in host immune response pathways without deamination but interact with viral proteins, viral RNAs, or other host immune factors [24–26]. Regardless of whether deamination is dependent or independent, host-mediated RNA editing can substantially influence the viral life cycle, host adaptation, or evolutionary directions to some extent [21, 27, 28]. Moreover, host-mediated RNA editing can lead to either proviral or antiviral effect on viruses [29], corresponding to increased viral fitness or diminished viral growth, respectively. Specifically, the proviral effect helps viruses evade the host immune response through promoting virus replication [30] and protein synthesis [31] as well as reducing virus toxicity [32]. Contrastingly, the antiviral effect works against a broad range of viruses through inhibition of virus replication [33] or reverse transcription [34].

**Fig. 2:**
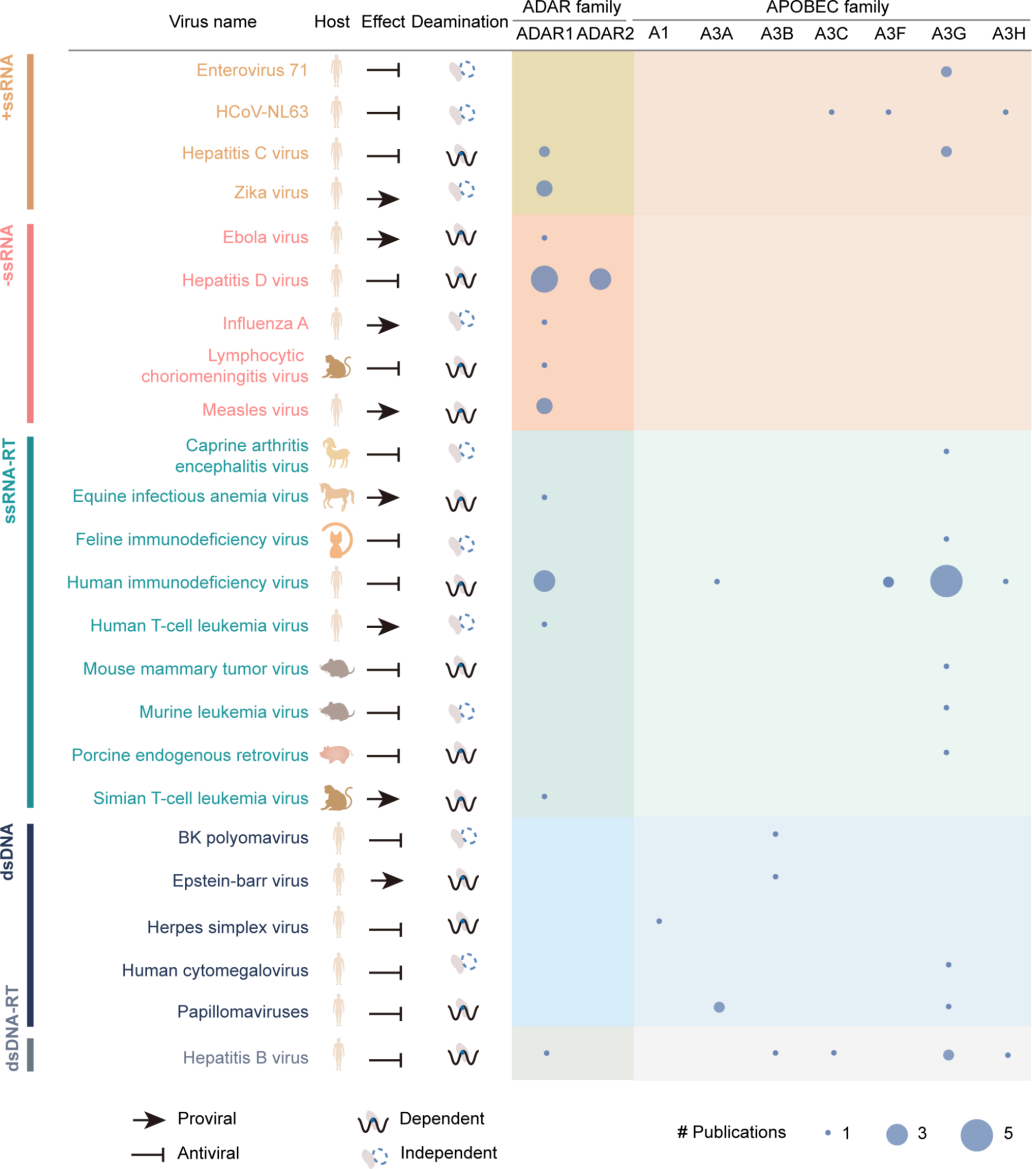
The landscape of editing mechanisms (deamination dependent/independent) and effects (proviral/antiviral) in viruses with experimental support. All relevant editing enzymes are listed together with the number of supported publications

To date, a wealth of RNA editing sites have been detected in different classes of viruses except single-stranded DNA (ssDNA) and double-stranded RNA (dsRNA) viruses according to the Baltimore Virus Classification. In this study, we summarize the research progresses of host-mediated RNA editing with experimentally validated evidence by grouping viruses into positive-sense single-stranded RNA (+ ssRNA), negative-sense single-stranded RNA (-ssRNA), single-stranded RNA reverse transcribing (ssRNA-RT), double-stranded DNA (dsDNA), and double-stranded DNA reverse transcribing (dsDNA-RT), and accordingly delineate a landscape of the involved RNA editing mechanisms (deamination dependent/independent) and molecular effects (proviral/antiviral) between viruses and hosts (Fig. 2).

## RNA editing in + ssRNA viruses

**Fig. 3:**
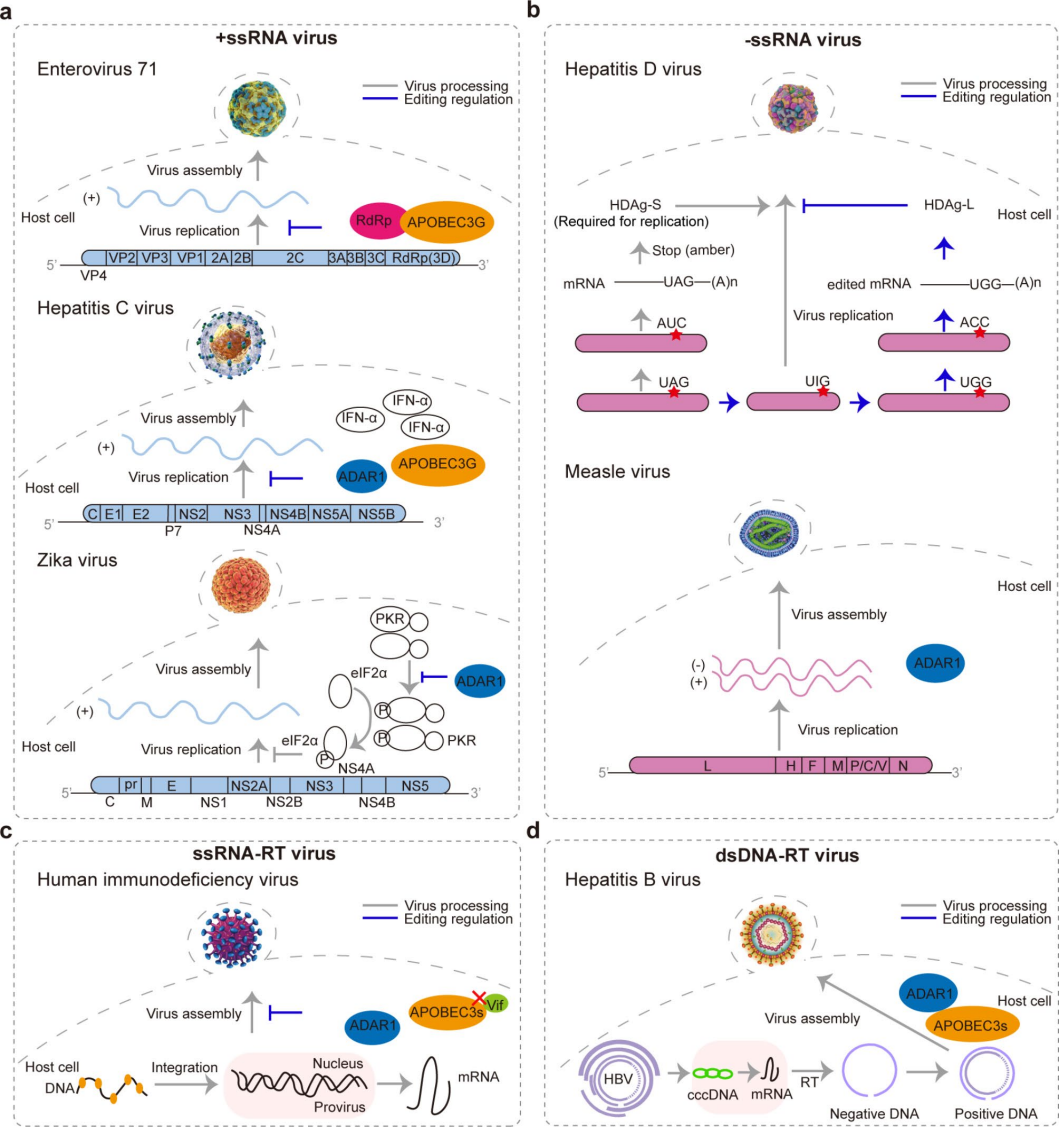
Biological processes of host-mediated RNA editing in viruses, supported with more than two independent publications. **(a)** RNA editing in + ssRNA virus. **(b)** RNA editing in -ssRNA virus. **(c)** RNA editing in ssRNA-RT virus. **(d)** RNA editing in dsDNA-RT virus

+ssRNA viruses infecting cellular hosts rely upon positive-stranded RNA as their primary genetic material [35]. Hitherto, host-mediated RNA editing sites have been documented in several + ssRNA viruses, for instance, enterovirus 71 (EV71) [36–40], hepatitis C virus (HCV) [24, 41–45], and zika virus (ZIKV) [30, 46–50]. In such viruses, both ADARs and APOBECs play important roles in the innate immune response commonly less dependent on deamination (Fig. 3A).

EV71, first described in 1974, is a kind of + ssRNA viruses that belongs to the *Picornaviridae* family, with genome size of about 7.4 kb nucleotides. EV71 can target the human central nervous system and cause hand-foot-mouth disease and herpangina, commonly in children under 5 years old [51]. Multiple lines of evidence have shown that APOBEC3G, a host restriction factor of EV71, exerts an antiviral effect by inhibiting virus replication and infectivity without requiring deamination activity [36, 37]. First, the change of key deaminated residues in APOBEC3G (H257R and E259Q in the C-terminal of CD2 domain) does not affect its antiviral effect [36], indicating that the single-stranded RNA-binding domain, but not the deamination activity of APOBEC3G, is essential for antiviral effect [37]. Second, although non-structural protein 2C of EV71 can antagonize APOBEC3G’s suppression, replication of EV71 is slowed down in APOBEC3G-expressed cells because APOBEC3G could competitively bind to EV71’s 5’UTR and accordingly inhibit EV71 protein synthesis [37]. Third, the infectivity of EV71 can also be reduced by APOBEC3G due to its interaction with virus protein 3D (also known as RdRp, assisting the synthesis of EV71) and packaging into progeny virions [36]. Thus, RNA editing acting on EV71 is associated with APOBEC3G in favor of antiviral effect without deamination.

HCV, first isolated in 1989, is a member of the *Flaviviridae* family with genome size of about 9.6 kb nucleotides [52]. It is capable to infect hepatocytes and extrahepatic cells, causing chronic liver disease, cirrhosis, and even hepatocellular carcinoma [53]. Studies have shown that both ADAR1 and APOBEC3G have antiviral effects on HCV yet through different mechanisms [24, 43, 45]. In detail, ADAR1 is identified as an important contributor to the innate immune response (including dsRNA-dependent protein kinase, PKR, and interferon, such as IFN-α) during HCV life cycle [24], which is supported by the finding that IFN-α may specifically restrain HCV replication through A-to-I editing [45]. Moreover, two polymorphism sites in ADAR1 (viz., rs1127326 and rs2229857) are significantly associated with the outcome of HCV clinical therapy [24] and the depletion of ADAR1 can enhance HCV replication [24]. While, for APOBEC3G, its expression is reported to be elevated in hepatocytes of HCV-infected patients [43] and knockdown experiments revealed its role on the inhibition of HCV replication without hypermutation in the viral genome [44], indicating APOBEC3G’s antiviral effect on HCV without requiring deamination.

ZIKV, with genome size of about 10.8 kb nucleotides, was first isolated in 1947 from *rhesus macaque* [54]. As a member of the *Flaviviridae* family, it can be transmitted through blood, placenta, and sex [55], and cause severely abnormal nervous diseases, such as congenital Zika syndrome and Guillain-Barré syndrome in children and adults, which might be explained by one hypothesis that abnormal RNA editing events mediated by host ADAR1 are associated with disease pathogenesis as a role of host immune mechanisms [47]. Different from HCV, host-mediated RNA editing exerts a proviral effect on ZIKV, which is critical to ensuring viral life activities [50]. Specifically, ADAR1 promotes ZIKV replication by preventing the phosphorylation of eIF2α carried out by PKR and IFN during the innate antiviral immune response and by decreasing host cell apoptosis to a certain degree [30]. This proviral effect is mediated by both ADAR1p150 (a full-length interferon-inducible isoform of ADAR1 that mainly localizes to the cytoplasm) and ADAR1p110 (a shorter and constitutively active isoform that resides in the nucleus) [30]. Of note, the deamination activity is unnecessary for this proviral effect since mutations in the deaminase domain of ADAR1p150 and ADAR1p110 do not affect the viral replicon RNA [30].

**Fig. 4:**
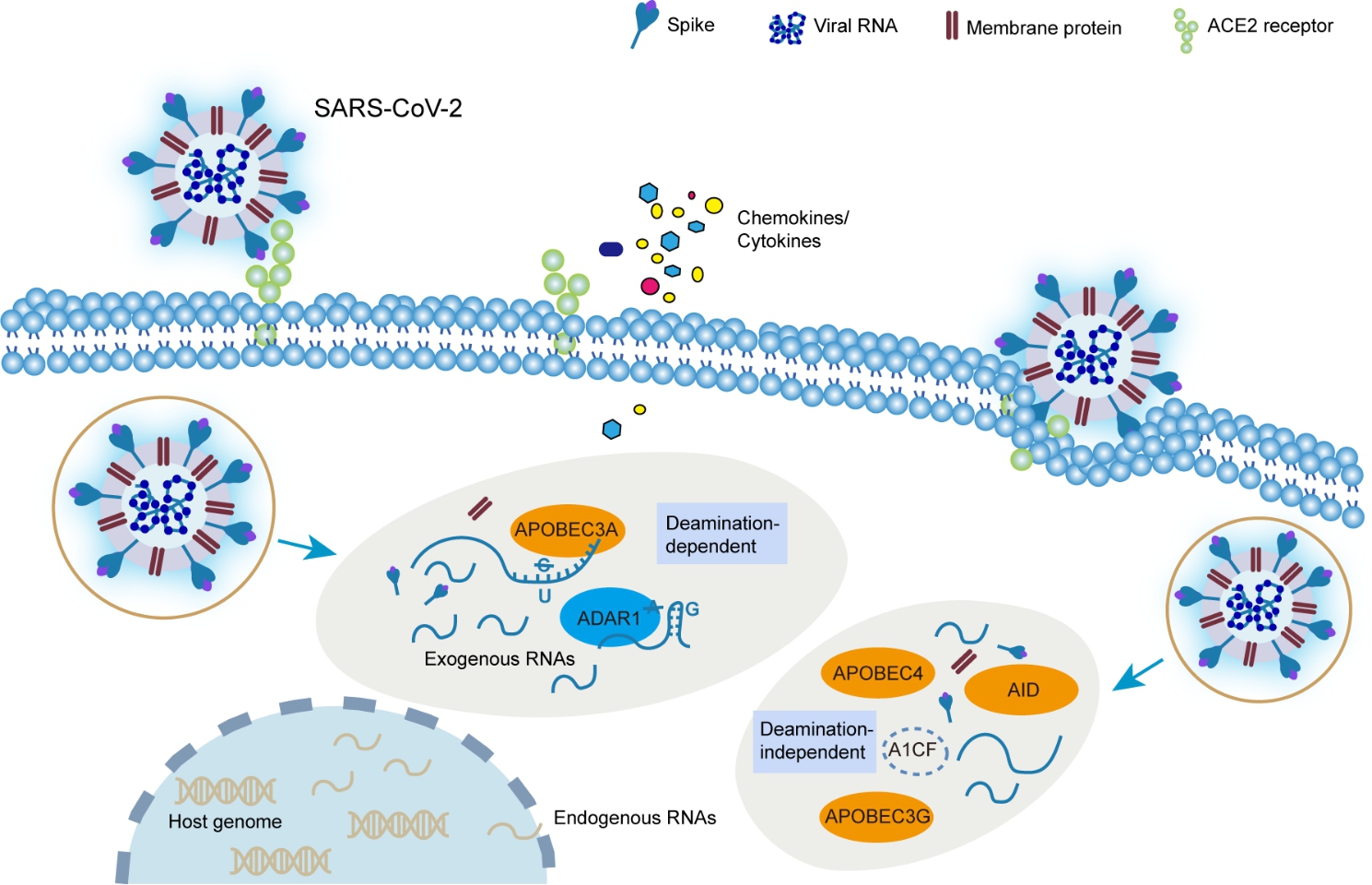
Schematic overview of the potential role of APOBECs and ADARs in host-mediated RNA editing of SARS-CoV-2. In SARS-CoV-2, both ADAR1 and APOBEC3A are associated with exogenous viral RNAs through deamination-dependent mechanism. By contrast, other members of APOBEC family, such as AID, APOBEC3G, and APOBEC4, together with partners (like A1CF), exert effects through deamination-independent mechanism

SARS-CoV-2, as a member of + ssRNA viruses belonging to the *Coronaviridae* family with the largest genome (~ 30 kb) among RNA viruses, caused the global pandemic due to its highly adaptive genomic variants [56, 57]. Albeit absent from adequate experimental evidence, integrative bioinformatic analysis of large-scale RNA-seq data revealed potential C-to-U and A-to-I conversions mediated by APOBECs and ADARs along the SARS-CoV-2 genome [58–62] (Fig. 4). It has also been reported that both APOBECs and ADARs may function in host immune response pathways to exert different effects on SARS-CoV-2 [63–65] (Fig. 4). Among the APOBECs, AID is the key initiation factor of the host humoral immune response against SARS-CoV-2 [66], while APOBEC1 and APOBEC3A, especially APOBEC3A, are likely to be responsible for C-to-U transformation and make a proviral effect by facilitating replication and propagation of SARS-CoV-2 [67]. Moreover, APOBEC-1 complementation factor (A1CF), a partner of APOBEC1, can interact with SARS-CoV-2 RNAs directly and specifically [68]. In addition, APOBEC4 is up-regulated in patients infected with SARS-CoV-2, causing a host antiviral response [69]. As for the ADAR members, ADAR1 may mediate A-to-I conversion and inhibit SARS-CoV-2 replication [60, 70] with an antiviral effect [62, 71].

## RNA editing in -ssRNA viruses

Unlike + ssRNA viruses, the genetic materials of -ssRNA viruses consist of single-stranded RNAs of the negative or antisense strand that do not encode proteins [72]. To date, RNA editing has been found to exert influences on -ssRNA viruses, such as hepatitis D virus (HDV) [19, 21, 73–92] and measles virus (MeV) [93–95] (Fig. 3B). Generally, in contrast to + ssRNA viruses, -ssRNA viruses seem to be more subjected to deamination by ADARs, which have an antiviral effect, especially in HDV.

HDV, belonging to the *Kolmioviridae* family, is a circular-RNA virus of ~ 1.7 kb nucleotides and its life cycle requires hepatitis B virus (HBV) for assembly and release of HDV particles [75]. Infection of HDV can cause liver diseases, such as chronic or severe hepatitis and cirrhosis. It was revealed that ADAR1 plays a central role in the inhibition of virus replication [19, 90]; ADAR1 could catalyze HDV amber/W editing site, which was first identified as U-to-C [19] and later corrected as A-to-G [90] due to the discovery of HDV circular antigenomic RNA. Due to A-to-G editing on amber/W site, two variants of hepatitis delta antigen (HDAg), namely, HDAg-S and HDAg-L, are produced [80, 96, 97]. Specifically, the original codon “UAG” stops the translation of HDAg and produces HDAg-S that can promote virus replication, whereas the edited codon “UGG” encodes tryptophan and yields HDAg-L that can inhibit virus replication. Thus, ADAR1 provokes an antiviral effect after HDV infection by producing HDAg-L [92]. During this process, ADAR1p110 plays a major role through deamination-dependent activity [85], while ADAR1p150 is responsible for IFN-α stimulation without deamination [83, 84]. Aside from ADAR1, it was proven that the HDV amber/W site can also be catalyzed by ADAR2 but with much lower editing efficiency [21].

MeV, belonging to the *Paramyxoviridae* family with genome size of about 15.9 kb nucleotides, is thought to emerge in the 6th century BCE (Before Common Era) [98]. Infection with MeV can cause measles, resulting in conjunctivitis, koplik spots, and rash [98]. It has been reported that ADAR1, especially ADAR1p150, exhibits a proviral effect by enabling MeV to evade host antiviral immunity and acts as an antiapoptotic host factor during MeV infection without requiring catalytic activity [93]. Further evidence indicates that MeV replication can be restricted in ADAR1^KO^ cells [94] and the damage on the virus caused by the innate immune response is associated with ADAR1 [95].

Aside from HDV and MeV, RNA editing has also been reported in several other –ssRNA viruses, such as influenza A virus (IAV) [99, 100], dengue virus [99], lymphocytic choriomeningitis virus (LCMV) [101], Ebola virus (EBOV) [102], and Marburg virus [102]. It was computationally suggested that host-mediated RNA editing is essential for IAV genome replication and viral protein synthesis [99], exerting a proviral effect by ADAR1p150 to activate the RIG-I (retinoic acid inducible gene I) signaling pathway without deamination [100]. It was also found in dengue virus, in which ADAR1 enhances virus replication by promoting non-structural protein synthesis [99]. Similarly, infection of LCMV upregulates ADAR1 expression and induces A-to-G mutations, which ultimately inhibit viral protein function and infectivity [101]. Moreover, EBOV and Marburg virus can be modulated by ADAR1 through A-to-I transversion in 3’-UTR, which results in negative regulation of virus translation [102].

## RNA editing in ssRNA-RT viruses

ssRNA-RT viruses can establish infection through integrating a DNA copy of the viral genome into host cell chromosome [103]. Till now, studies have discovered that several ssRNA-RT viruses are subjected to host-mediated RNA editing by ADAR1 and APOBECs through deamination-dependent/independent activity [14, 15, 25, 34, 104–123] (Fig. 3C).

Human immunodeficiency virus (HIV), first isolated in 1983 from human, is an enveloped RNA retrovirus with genome size of about 9.2 kb nucleotides [124] that can render acquired immune deficiency syndrome (AIDS). Both ADARs and APOBECs are involved in the host-mediated RNA editing on HIV (including HIV-1 and HIV-2) [108, 121, 122]. On the one hand, ADAR1 affects HIV in a proviral manner by deaminating adenosines [104–108, 120] in the 5’-UTR to stimulate viral infection [108], interacting with PKR to promote virus replication during infection of lymphocytes [120], and enhancing the expression of VP24 (one of HIV proteins) [107]. On the other hand, ADAR1 can also act as an antiviral factor inhibiting viral infectivity and protein synthesis [105]. Unlike the dual effects of ADAR1, APOBEC3s, including APOBEC3A [111], APOBEC3F [112], APOBEC3G [116], and APOBEC3H [116], are mainly involved in inhibiting the replication of both HIV-1 and HIV-2 [121, 122]. Among them, APOBEC3G is well studied to be able to interact with HIV-1 reverse transcriptase and inhibit HIV-1 replication without deamination [34, 125]. Strikingly, to antagonize the inhibition of APOBEC3G, the virion infectivity factor (vif) of HIV [25], a viral accessory protein, has been proven to interact directly with APOBEC3G [115] and prevent packaging of APOBEC3G into virion [123].

In addition to HIV, other ssRNA-RT viruses, albeit with limited experimental evidence, are also reported to be affected by host-mediated RNA editing through APOBEC3s and ADARs. It is indicated that life activity of caprine arthritis encephalitis virus (CAEV), feline immunodeficiency virus (FIV), murine leukemia virus (MLV), mouse mammary tumor virus (MMTV), and porcine endogenous retrovirus (PER) can be antagonized by host-mediated RNA editing (with or without requiring deamination), potentially owing to degradation of APOBEC3s by vif protein [15, 113, 114, 118, 119]. Specifically, in CAEV [113] and FIV [114], just like HIV, the antiviral effect of APOBEC3s can be counteracted by vif protein, glycosylated Gag (glyco-Gag) protein in MLV could protect the reverse transcription complex from APOBEC3s’ antagonization [119], and the reverse transcription and replication of MMTV and PER replicon RNAs can be inhibited by APOBEC3s through deamination activity [15, 118]. Different from APOBEC3s, ADAR1 mainly makes proviral effects on equine infectious anemia virus (EIAV), simian T-cell leukemia virus (STLV), and human T-cell leukemia virus (HTLV) [14, 109, 110]. It was reported that ADAR1 contributes to the adaptation of EIAV [14] and STLV [110] by increasing A-to-G editing level through deamination. Moreover, ADAR1 also promotes HTLV replication by inhibiting the host PKR activity without requiring deamination [109].

## RNA editing in dsDNA viruses

In addition to RNA viruses, dsDNA viruses, such as BK polyomavirus (BKPyV), Epstein-Barr virus (EBV), human cytomegalovirus (HHV), human papillomaviruses (HPV), and herpes simplex virus (HSV), have been reported to be targeted and impacted by host-mediated RNA editing, in which APOBECs are the main mediators [126–131]. For example, APOBEC3B, regarded as an innate immune DNA cytosine deaminase sensor, is upregulated after infection of both BKPyV and EBV, thereby activating host innate immunity against viruses [126, 127]. In BKPyV, APOBEC3B is influenced by virus large T antigen, leading to the activation of immune factors like interferon-stimulated genes [126, 127]. In EBV, knockdown of BORF2, the large subunit of the viral ribonucleotide reductase, can activate APOBEC3B and further cause lower viral load, infectivity, and hypermutation [126, 127]. Moreover, it was revealed that APOBEC3G also plays an antiviral effect on HHV [129] and HPV [128] by inhibiting virus infection. Aside from APOBEC3s, APOBEC1 might also exert an antiviral effect with deamination that further reduces virus replication on HSV [130]. Remarkably, C-to-T transitions on HSV *UL54* gene through APOBEC1 deamination activity are essential impaired accumulation of HSV DNA copy numbers and mRNA transcripts [130].

To be specific, studies have documented that host-mediated RNA editing influences viral load of HPV [128, 131] that is the chief culprit of cervical cancer. APOBECs, especially APOBEC3A and APOBEC3G, participate in regulating cell viability [128, 131]. In detail, APOBEC3A exerts an antiviral effect caused by C-to-T transitions of HPV *E6* gene [131]. Consistently, it was also reported that knockdown of APOBEC3A or APOBEC3G reduces the frequency of A + T in *E2* gene of HPV [128]. In total, APOBECs are inclined to play important roles in host-mediated RNA editing by deamination-dependent activity in dsDNA viruses.

## RNA editing in dsDNA-RT viruses

dsDNA-RT viruses, albeit well studied with respect to innate immunity [132], have limited evidence in host-mediated RNA editing [133]. HBV, with a genome size of about 3.2 kb nucleotides, is the first human hepatitis virus from which the proteins and genome could be identified and characterized [134]. In human, HBV infection can cause acute and chronic liver diseases, including cirrhosis and hepatocellular carcinoma [134], which are related with immunomodulatory factors, such as APOBECs, to induce innate and adaptive immune responses [135].

Evidence has shown that APOBECs can influence HBV replication by complex host-mediated RNA editing mechanisms (Fig. 3D). First, reverse transcription, a part of dsDNA-RT viral replication process, is inhibited by APOBEC3B through deamination [136–138]. In addition, RNA binding proteins, like DHX9, can reduce the antiviral effect of APOBEC3B as co-factors [136–138]. Second, APOBEC3C, APOBEC3G, and APOBEC3H also contribute to the innate anti-HBV immune response in host [139, 140]; in human liver, the three catalytic proteins inhibit HBV viral replication by hypermutation of DNA sequences [139, 140]. On the contrary, APOBEC3F exerts deamination-independent activity through IFN-α release and is also involved in HBV replication [141]. Notably, APOBEC3G inhibits HBV replication, especially interference of reverse transcription by deamination-dependent activity [142–144]. Meanwhile, it also inhibits HBV reverse transcription without deamination, targets viral DNA-RNA hybrids [145] and is involved in the innate antiviral immune response [146].

## Concluding remarks & perspectives

Through synthesizing the current knowledge of host-mediated RNA editing in a variety of viruses, it is clearly shown that RNA editing can induce proviral or antiviral effects by promoting or inhibiting virus replication, protein synthesis and/or infectivity with or without requiring deamination. According to the synthesized knowledge, deamination-dependent RNA editing is mainly found in -ssRNA viruses, whereas deamination-independent regulation is observed in the majority of + ssRNA viruses, indicating their divergence in RNA editing mechanisms. This is presumably due to the difference in viral replication mechanism and life cycle, as the genetic materials of + ssRNA viruses could be translated as mRNA, while -ssRNA viruses need additional steps for transcription/reverse transcription [72, 103]. Specifically, during the additional steps, complementary RNAs of -ssRNA viruses are transcribed as intermediates, forming dsRNA substrates that may be easier to be edited directly by ADAR1. Moreover, the preference of deamination-dependent/independent RNA editing mechanism may be also related to viral transmission routes. Deamination-independent mechanism prefers respiratory and digestive systems for virus transmission, whereas deamination-dependent mechanism prefers blood system transmission. Furthermore, different classes of viruses present differences and similarities in terms of host-mediated RNA editing (Fig. 2). Noticeably, RNA viruses prefer to employ both ADARs and APOBECs, whereas DNA viruses tend to use APOBECs only, which will be enriched, updated, and evolved with future related studies. APOBECs, particularly APOBEC3G, exert antiviral effects in nearly all viruses except -ssRNA viruses, while ADARs participate in nearly all viruses except dsDNA viruses. In addition, many other characteristics, such as hosts [14] and virus toxicity [32], may be also associated with RNA editing mechanisms. Collectively, these results indicate the diversity of RNA editing between hosts and viruses, yet requiring more experimental investigations to decipher complex underlying mechanisms.

As also indicated in this report, host endogenous RNAs and exogenous viral RNAs could be distinguished by differential editing patterns mediated by ADAR enzymes [147]. Endogenous RNAs are appropriately edited, while invasive exogenous viral RNAs are edited at low levels upon infection. High abundance of lowly edited viral RNAs with dsRNAs could lead to the activation of dsRNA sensor (MDA5) and subsequent antiviral interferon response pathway [148]. Thus, aberrant RNA editing in viral RNAs may be beneficial to the immune escape of virus, due to the loss of capability of host cell to discriminate endogenous RNAs from exogenous viral RNAs [147].

Host-mediated RNA editing, albeit relatively weak in viruses, may be an important contributor to virus evolution [149]. It has been found that viruses can reduce toxicity, enhance infectivity, and speed up replication by changing viral core genetic sequences through host-mediated RNA editing mechanisms [21, 73]. It is believed that RNA editing events in different virus clades might be under differential evolutionary selection and contribute to viral phenotypic diversity [57]. Moreover, specific non-synonymous editing sites, like amber/W site in HDV, are considered as critical events provoking proviral or antiviral effects [90]. Thus, host-mediated RNA editing may act as a cradle to foster the adaptive evolution of virus, which is yet heterogenous in different classes of viruses. One hypothesis is that deamination-dependent activity contributes to the adaptive evolution of -ssRNA viruses, which needs to be further investigated, both computationally and experimentally.

Undoubtedly, existing studies on RNA editing in viruses have limitations. RNA editing sites in viruses can be missed or miscalculated due to low sequencing coverage, sequencing error or/and short read length. Specifically, as multiple RNA editing sites may be co-edited and exert complex effects, it is challenging to identify/verify the coexistence of adjacent RNA editing sites based on short sequencing reads, especially for exogenous RNAs. In the future, long reads generated by the third-generation sequencing technology have great potential to capture a complete picture of RNA editing sites and to detect cooperativity among RNA editing sites during dynamic viral life cycles.

In addition, given the ongoing pandemic of COVID-19, it would be desirable to conduct a SARS-CoV-2 cohort study to systematically investigate the host-mediated RNA editing mechanisms and molecular effects on SARS-CoV-2 and further explore the editing heterogeneity among infected people with mild, moderate and severe symptoms. Notably, a model of RNA editing on SARS-CoV-2 transcriptome was proposed [65]; although this model explains differential editing frequencies and patterns before and after viral replication on positive- and negative-sense viral transcripts, it cannot elucidate diverse effects of RNA editing on SARS-CoV-2 in patients with different symptoms.

Taken together, our study delineates the landscape of RNA editing-associated mechanisms and effects in different classes of viruses, which would provide potentially valuable insights and also call on worldwide collaborative efforts, particularly during the pandemic caused by SARS-CoV-2 as well as monkeypox virus, for better understanding the host-mediated RNA editing on both ever-reported and newly-emerging viruses.

## Data Availability

Not applicable.
